# Site-Specific Glycosylation Patterns of the SARS-CoV-2 Spike Protein Derived From Recombinant Protein and Viral WA1 and D614G Strains

**DOI:** 10.3389/fchem.2021.767448

**Published:** 2021-11-19

**Authors:** Yuan Tian, Lisa M. Parsons, Ewa Jankowska, John F. Cipollo

**Affiliations:** Food and Drug Administration, Center for Biologics Evaluation and Research, Division of Bacterial, Parasitic and Allergenic Products, Silver Spring, MD, United States

**Keywords:** SARS-CoV-2, N-glycosylation, O-glycosylation, glycan shield, cell substrate, spike protein, microheterogeneity, D614G variant

## Abstract

The SARS-CoV-2 spike protein is heavily glycosylated, having 22 predicted N-glycosylation sites per monomer. It is also O-glycosylated, although the number of O-glycosites is less defined. Recent studies show that spike protein glycans play critical roles in viral entry and infection. The spike monomer has two subdomains, S1 and S2, and a receptor-binding domain (RBD) within the S1 domain. In this study, we have characterized the site-specific glycosylation patterns of the HEK293 recombinant spike RBD and S1 domains as well as the intact spike derived from the whole virus produced in Vero cells. The Vero cell-derived spike from the WA1 strain and a D614G variant was analyzed. All spike proteins, S1, and RBDs were analyzed using hydrophilic interaction chromatography (HILIC) and LC-MS/MS on an Orbitrap Eclipse Tribrid mass spectrometer. N-glycans identified in HEK293-derived S1 were structurally diverse. Those found in the HEK293-derived RBD were highly similar to those in HEK293 S1 where N-glycosites were shared. Comparison of the whole cell-derived WA1 and D614G spike proteins revealed that N-glycosites local to the mutation site appeared to be more readily detected, hinting that these sites are more exposed to glycosylation machinery. Moreover, recombinant HEK293-derived S1 was occupied almost completely with complex glycan, while both WA1 and D614G derived from the Vero E6 cell whole virus were predominantly high-mannose glycans. This stands in stark contrast to glycosylation patterns seen in both CHO- and HEK cell-derived recombinant S1, S2, and the whole spike previously reported. Concerning O-glycosylation, our analyses revealed that HEK293 recombinant proteins possessed a range of O-glycosites with compositions consistent with Core type 1 and 2 glycans. The O-glycosites shared between the S1 and RBD constructs, sites T323 and T523, were occupied by a similar range of Core 1 and 2 type O-glycans. Overall, this study reveals that the sample nature and cell substrate used for production of these proteins can have a dramatic impact on the glycosylation profile. SARS-CoV-2 spike glycans are associated with host ACE2 receptor interaction efficiency. Therefore, understanding such differences will serve to better understand these host–pathogen interactions and inform the choice of cell substrates to suite downstream investigations.

## Introduction

SARS-CoV-2 is an enveloped, positive single-stranded RNA beta coronavirus expressing four main structural proteins, which include nucleocapsid, spike, membrane, and envelope proteins (Lu et al., 2020; Singh and Yi, 2021). The trimeric spike protein is the major surface protein of the SARS-CoV-2 virus and serves as an entry protein for host cell infection (Shang et al., 2020). To facilitate the fusion of the viral membrane with the infected cells, the spike proteins are cleaved into S1 and S2 subunits by cellular proteases, such as furin (Hoffmann et al., 2020a; Hoffmann et al., 2020b; Xia, 2021). The S1 subunit contains the N-terminal and the receptor-binding domain (RBD) (Xia, 2021), and recombinant RBD binds to the human angiotensin converting enzyme 2 (ACE2) present as a surface receptor on host cells. The S2 domain serves the function of membrane fusion, which contains a fusion peptide (FP), an internal fusion peptide (IFP), two heptad repeat domains (HR1 and HR2), a transmembrane domain, and a C-terminal domain (Coutard et al., 2020; Xia, 2021).

Enveloped viruses have evolved to take advantage of many host cell processes including glycosylation (Watanabe et al., 2019). Viral protein glycosylation functions in a number of ways in the viral lifestyle including viral particle stability, mediating viral infection, host immune response, and immune evasion (Walls et al., 2016; Watanabe et al., 2019). Viral glycosylation of key envelope glycoproteins can be dynamic over time as the virus propagates through the host population, allowing immune avoidance to evolve over time (Li et al., 2021). Recent cryo-EM studies reported that the recombinant SARS-CoV-2 spike protein is extensively glycosylated (Grant et al., 2020; Wrapp et al., 2020). Using recombinant proteins, earlier studies reported glycosylation of the 22 predicted N-linked glycosites in the spike protein at high occupancy and lower glycosylation occupancy on O-linked glycosites (Watanabe et al., 2020a; Watanabe et al., 2020b; Shajahan et al., 2020; Zhao et al., 2020; Zhou et al., 2021). A recent study reported that glycosylation is essential for SARS-CoV-2 viral entry and infection (Yang et al., 2020). Since glycans are produced through a stochastic process that is dependent upon glycosylation, enzyme expression, location, concentration, and the particular glycoprotein’s sequence and structural characteristics, it can be altered under selective pressure. During viral evolution, with passage through the human population, glycosites are added and deleted often, leading to an increased number of sites and glycan complexity. The overall glycosylation characteristics such as composition, subclass, heterogeneity, and density over the surface of the protein can have dramatic effects on viral survival, transmission, and immune evasion (Vigerust and Shepherd, 2007; Watanabe et al., 2019; Li et al., 2021). Spike glycoproteins are often the major target for vaccine design and antivirus drug development. Understanding the glycosylation microheterogeneity of the spike protein can facilitate the process.

Here, we characterize site-specific glycosylation on recombinant RBD and the S1 domain of the spike protein produced in HEK293 cells to understand the glycosylation microheterogeneity produced using this cell substrate. The question remains open: whether the glycosylation of these recombinant proteins differs from that of the native spike produced in the whole virus. Thus, we compare the glycosylation of recombinant RBD and S1 to two intact viruses, the WA1 strain and a D614G variant, both produced in Vero E6 cells. The SARS-CoV-2/USA-WA1/2020 (USA-WA1) viral strain was isolated from the specimen of the first confirmed case in the United States (Harcourt et al., 2020; Wang et al., 2021a). Whole genome sequencing confirmed that this strain contained D614 as the original form of the SARS-CoV-2 virus. SARS-CoV-2/Massachusetts/VPT1/2020 (MA/VPT1), containing the D614G mutation, was isolated in Vero E6 cells from a nasopharyngeal specimen collected in April 2020 (Wang et al., 2021a). The D614G mutation, which appeared in early 2020 (Korber et al., 2020), has become dominant worldwide. The D614G mutation is also carried by the more recent and concerning SARS-CoV-2 variants, including B.1.1.7, B.1.351, P.1, and B.1.617 (https://www.cdc.gov/coronavirus/2019-ncov/variants/). Compared to strains containing the original D614, viruses with the D614G mutation have significantly higher infection titers as well as faster transmission but are less sensitive to spike-based SARS-CoV-2 vaccine sera produced in mice, non-human primates, and humans (Hou et al., 2020; Korber et al., 2020; Yurkovetskiy et al., 2020). In addition, structural analysis demonstrates that the G614 spike is in a more open conformation with extended RBDs (Yurkovetskiy et al., 2020). Given this conformational shift, it is of interest to examine glycosylation for possible changes in the D614G spike compared to its close progenitor, the WA1 strain, while keeping the viral propagation cell platform the same. Therefore, in addition to analysis of recombinant spike constructs, we report the glycosylation patterns of spikes in WA1 and D614G strains produced by the whole virus in Vero E6 cells. Our results may aid in interpretation of experimental data concerning spike interactions with the host and surrogates as well as the development of therapeutics and vaccines against the SARS-CoV-2 virus.

## Materials and Methods

### Recombinant Proteins and Intact Viruses

Recombinant protein SARS-CoV-2 Spike S1 and RBD proteins expressed in HEK293 cells were purchased from Sanyou Bio (China). The whole virus of the WA1 strain was from the first patient of SARS-CoV-2 virus infection in the United States (Harcourt et al., 2020). The virus was isolated from nasopharyngeal and oropharyngeal specimens from this patient, and the viral sequence was confirmed (Wang et al., 2021a). This strain, SARS-CoV-2/USA-WA1/2020 (USA-WA1), is the original form of the SARS-CoV-2 virus without mutation at the 614 amino acid (Li et al., 2021). The D614G variant carrying the spike protein amino acid change at 614D to G, SARS-CoV-2/Massachusetts/VPT1/2020 (MA/VPT1), was isolated from Vero E6 cells from a nasopharyngeal specimen collected in April 2020 (Wang et al., 2021a). Both viruses were grown in Vero E6 cells, and the supernatant of the passage 4 stock of each virus was collected by centrifugation. After the viruses were frozen to −80°C at least overnight, the viruses were inactivated by gamma irradiation.

Chemicals, reagents, and TSKgel amide 80 particles were purchased from Tosoh Bioscience LLC (Montgomeryville, PA). Sep-Pak C18 cartridges were purchased from Waters (Milford, MA, United States). Sequencing grade-modified trypsin, chymotrypsin, and Glu-C were purchased from Promega Corp. (Madison, WI). PNGase F was purchased from New England BioLabs, Inc (Ipswich, MA). Iodoacetimide, dithiothreitol (DTT), trifluoroacetic acid (TFA) (≥99%), and other chemicals were purchased from Sigma-Aldrich (St. Louis, MO, United States). Solvents were of high-pressure liquid chromatography (HPLC) grade or higher and purchased from Thermo Fisher Scientific (Waltham, MA). All other reagents were of ACS grade or higher.

### Protein Digestion

Recombinant proteins (200 µg) were dissolved in 50 mM ammonium bicarbonate at a concentration of 2 µg/µL. DTT was added to reduce the disulfide bonds at a final concentration of 5 mM for 30 min at 60°C. The samples were cooled to room temperature (RT), and iodoacetamide (IAA) was added to alkylate the reduced cysteine residues at a concentration of 15 mM for 30 min in the dark at RT. DTT was added to 25 mM to neutralize the remaining IAA. Trypsin or chymotrypsin was added (enzyme/protein, 1:50, w/w), and the samples were incubated at 37°C overnight.

For intact viruses, approximately 500 µg of the proteins was reduced with 5 mM DTT in 6M urea and 50 mM NH_4_HCO_3_ for 1 h at 37°C and subsequently alkylated with 15 mM iodoacetamide for 30 min at RT in the dark. To neutralize the remaining IAA, DTT was added to 25 mM. Samples were diluted 6-fold with 50 mM NH_4_HCO_3_ and 1 mM CaCl_2_ and digested with sequencing grade-modified trypsin or chymotrypsin at 1:50 (enzyme/protein, w/w) overnight at 37°C. Glu-C was added to the tryptic digest at 1:50 (enzyme/protein, w/w) and incubated overnight at 37°C.

### Glycopeptide Enrichment by the HILIC Resin

Intact glycopeptides were enriched by solid-phase extraction using the TSKgel amide 80 hydrophilic interaction chromatography (HILIC) resin according to our previous report (An and Cipollo, 2011). Briefly, 200 mg (400 µL of the wet resin) of the amide 80 resin was placed into a Supelco fritted 1 ml column and washed with 1 ml of 0.1% trifluoroacetic acid (TFA)–water solution. The column was conditioned with 1 ml of 0.1% TFA–80% ACN. The peptides were suspended in 0.1% TFA–80% ACN and slowly loaded to the column. The hydrophobic species were washed away with 3 ml of 0.1% TFA–80% ACN. For recombinant proteins, the glycopeptides were eluted with 1 ml of 0.1% TFA–50% ACN and 1 ml of 0.1% TFA–25% ACN. The eluents were combined and vacuum-dried. For whole viruses, the glycopeptides were eluted sequentially with 1 ml of 0.1% TFA–65% ACN, 0.1% TFA–60% ACN, 0.1% TFA–50% ACN, and 0.1% TFA–25% ACN. Each eluent was vacuum-dried and analyzed by mass spectrometry separately.

### Reversed-Phase HPLC Fractionation

The PNGase F-treated WA1 and D614G peptides were dried by speed vacuum and resuspended in 20 μl of 10 mM TEAB. The fractionation of the peptide samples is carried out using an Agilent Poroshell 120 Column (2.7 μm, 2.1 × 150 mm) and an Agilent UHPLC 1290 system. The separation was performed by running a gradient of Solvent B (10 mM TEABC, pH 8.0, 90% ACN) and Solvent A (10 mM TEAB, pH 8.0) at a flow rate of 200 μl/min in a 150 min run. The elute fractions are collected into a 96-well plate using a 1260 series auto-sample fraction collector. The 96 elute fractions were further combined into 12 fractions according to the collection time (combined per column into one fraction, 12 column 12 fractions). Each fraction was dried by rotary evacuation.

### Site Occupancy Analysis

Digested peptides were deglycosylated with PNGase F in 50 mM NH_4_HCO_3_. PNGase F cleaves between the innermost N-linked core GlcNAc and the Asn residue to which it is covalently linked. PNGase F deamidates the N-linked Asn producing an Asp residue, with a resulting increase of 0.984 Da in molecular weight (Gonzalez et al., 1992). PNGase F-treated peptides were desalted by C18 cartridge solid-phase extraction. The percent occupancy for each site is calculated by comparing the extracted chromatographic area under the curve of peptides with Asn to those with Asp using Byonic software (Version 3.10; Protein Metrics Inc.).

### Mass Spectrometry Analysis

The peptides were reconstituted in 0.1% formic acid–water solution and analyzed on an Orbitrap Eclipse Tribrid mass spectrometer equipped with a nanospray ion source and connected to a Dionex binary solvent system (Thermo Fisher Scientific). Peptides were separated using an Acclaim™ PepMap™ 100 C18 Column (75 μm × 15 cm). A trapping column (PepMap 100 C18 3 μM 75 μM × 2 cm) was used in line with the LC prior to separation with the analytical column. The solvent system consisted of solvent A (100% water/0.1% formic acid) and solvent B (100% ACN/0.1% formic acid). The LC conditions were as follows: 5–35% of solvent B for 165 min, 90% of solvent B for 5 min, and 1% of solvent B for 5 min. The flow rate was set to 300 nl/min. The spray voltage was set to 2.7 kV, and the ion-transfer tube temperature was set to 275°C. The full MS scan range was 400–2000 m/z. Precursor masses were detected in the Orbitrap at resolution (R) = 120,000 (at m/z 200). Stepped HCD (higher-energy collisional dissociation) spectra (HCD energy at 15, 25, and 35%) were recorded for the top 15 most abundant precursors with the standard mode of the AGC target. Dynamic exclusion was set at 30 s. If at least one typical glycan fragment ion abundance (m/z 204.0867 and 366.1396 Da) was observed within the top 15 most abundant fragments and within a 15 ppm mass accuracy, an EThcD [electron-transfer dissociation (ETD) followed by supplemental HCD collision energy at 25%] spectrum of the same precursor would be recorded in the Orbitrap at R = 15,000. The ETD reaction time was set to use calibrated charge-dependent ETD parameters. The glycopeptides of the intact virus were analyzed by stepped HCD fragmentation and HCD-triggered EThcD fragmentation to analyze N-linked glycans and O-linked glycans, respectively. Deamidated peptides were analyzed only by stepped HCD fragmentation.

### Data Analysis Using Byonic and Manual Verification

The LC-MS/MS spectra were searched against the FASTA sequence of the spike protein of the SARS-CoV-2 original virus or the D614G variant using Byos™ (Version 3.10; Protein Metrics Inc.). The searching parameters were specified as follows: fully specific digestion specificity, 2 missing cleavage sited allowed, carbamidomethyl at cysteine as a fixed modification, and oxidation at methionine as a common modification. The precursor ion mass tolerance was set at 6 ppm, and that for fragment ions was at 20 ppm. A 1% false discovery rate (FDR) was applied. The results were filtered with PEP 2D < 0.01, score ≥100, and Delta Mod. Score ≥10. The glycopeptide fragmentation data were evaluated manually for each glycopeptide; the peptide was confirmed when the b and y fragment ions were observed along with oxonium ions corresponding to the glycan identified. A minimum of 3 b/y ions were required. The relative amounts of each glycan at each site were determined by comparing the extracted chromatographic area under the curve. All charge states for a single glycopeptide were summed.

Glycans were categorized to subtypes according to the composition detected: Hex (4–10)HexNAc(2) was classified as high mannose, NeuAc (0–1)dHex (0–1)Hex (3–7)HexNAc(3) was classified as Hybrid, and NeuAc (0–5)dHex (0–3) Hex (3–8)HexNAc(4–7) was classified as complex-type glycans. Any glycan containing at least one fucose or sialic acid was counted as fucosylated or sialylated, respectively.

### Model Construction

Monomeric structural models of N-linked glycan presentation on SARS-CoV-2 were created using electron microscopy structures (PDB ID: 6ZGG), which were visualized with CCP4MG. Glycan cartoon structures are inferred from knowledge of common glycans as identification was done solely by mass. A trimeric structural model of SARS-CoV-2 was created from the electron microscopy structure (PDB ID: 7A96) and visualized with CCP4MG. The antigenic epitopes were predicted using NetCTL-1.2 (Larsen et al., 2007).

## Results and Discussion

### Mapping Glycosylation on Recombinant RBD Proteins

Recombinant RBD proteins expressed in HEK293 cells were trypsin-digested. Seventy-five percent of the digest was used for glycopeptide enrichment using HILIC separation, and 25% was deglycosylated in preparation for glycosylation site occupancy analysis. The HILIC-enriched intact glycopeptides were analyzed by LC-MS/MS using HCD-triggered EThcD fragmentation. The deglycosylated peptides were analyzed by LC-MS/MS with HCD fragmentation. The LC-MS/MS raw files were analyzed using Byonic software. The Byonic results were filtered with a 1% false discovery rate and other parameters to achieve high confidence identifications (see the *Method* section). All the spectra were manually verified.

**FIGURE 1:**
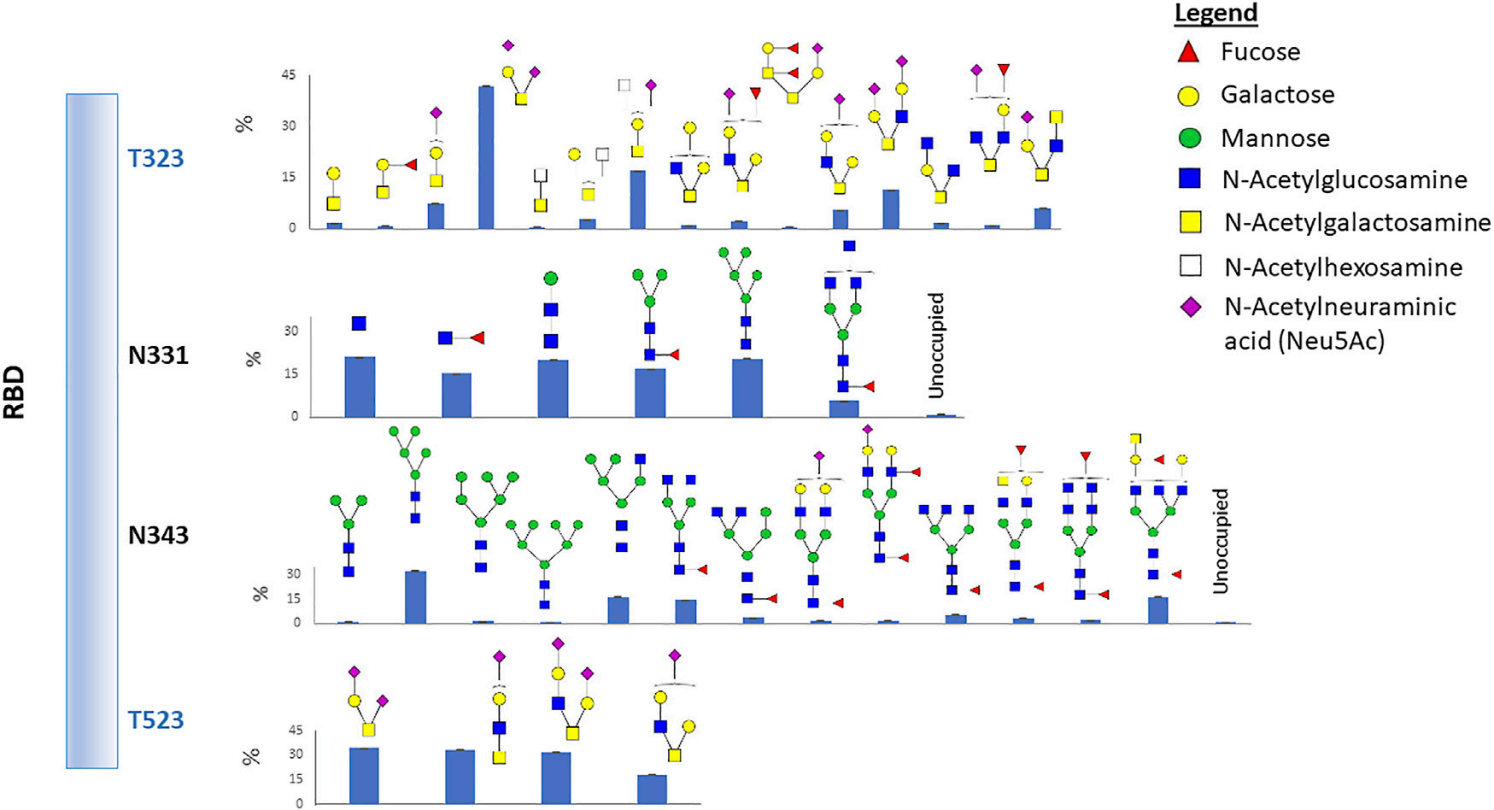
Glycosylation profile on the recombinant RBD protein. Two N-glycosites and two O-glycosites were identified. Glycan cartoon structures are inferred from knowledge of common glycans as identification was done solely by mass. The bar graphs represent the glycan abundance and unoccupied percent based on total ion abundances at each site.

The RBD has two potential N-linked glycosylation sites at amino acid positions 331 and 343 relative to the WA1 spike protein. Our data show that both sites are heavily glycosylated with greater than 99% occupancy (Figure 1 and Supplementary Table S1). We observed a high degree of fucosylation at the two N-glycosites, and Man5GlcNAc2 (Man5) is highly abundant at both sites (Figure 1 and Supplementary Table S1). Glycans identified at N331 included high mannose; short complex, paucimannose; and highly abbreviated forms (Figure 1; Supplementary Table S1 and Supplementary Figure S1). The reason is not fully understood but may be related to prompt fragmentation or degradative processes incurred during RBD production and/or purification. Prompt decay is unlikely as no other glycosites demonstrated this pattern.

We also identified two O-glycosylation sites at residues T323 and T523 with a diverse range of glycan compositions. Interestingly, most glycans at the two O-glycosites contain sialic acid (Figure 1 and Supplementary Table S2). Glycosylation of T323, but not T523, has been previously reported. Therefore, we carefully examined the spectra and observed strong evidence of glycosylation at T523 (Supplementary Figure S2). Previous studies reported O-glycosylation at T325 (Shajahan et al., 2020; Zhao et al., 2020), although the occupancy was estimated to be low (Zhao et al., 2020). However, our data did not show direct evidence of fragment ions which can confirm that T325 is glycosylated.

### Site-Specific Microheterogeneity of Spike Glycosylation in Recombinant S1 Proteins

The recombinant S1 protein expressed by HEK293 cells was treated according to the same protocol as the RBD protein (see above), except that two enzymes were used for digestion to facilitate glycoproteomic coverage of the protein. These two enzymes were trypsin and chymotrypsin, used in separate digestion. Byonic search parameters and filters were also the same as for the RBD protein.

The gene encoding the S1 domain has 13 possible sites of N-glycosylation. Twelve of the 13 predicted N-glycosites were found to be extensively glycosylated (Supplementary Figure S3 and Supplementary Table S3). The one missing glycosite, N17, was detected glycosylated, but it did not meet our criteria due to low confidence scores. Although the scores are low, many hybrid and complex-type glycans were detected at N17 with at least two technical replicates. The site occupancy for 10 glycosites is greater than 90%. Sites N149 and N657 had a site occupancy rate of 25 and 58%, respectively (Supplementary Figure S3 and Supplementary Table S3).

**FIGURE 2:**
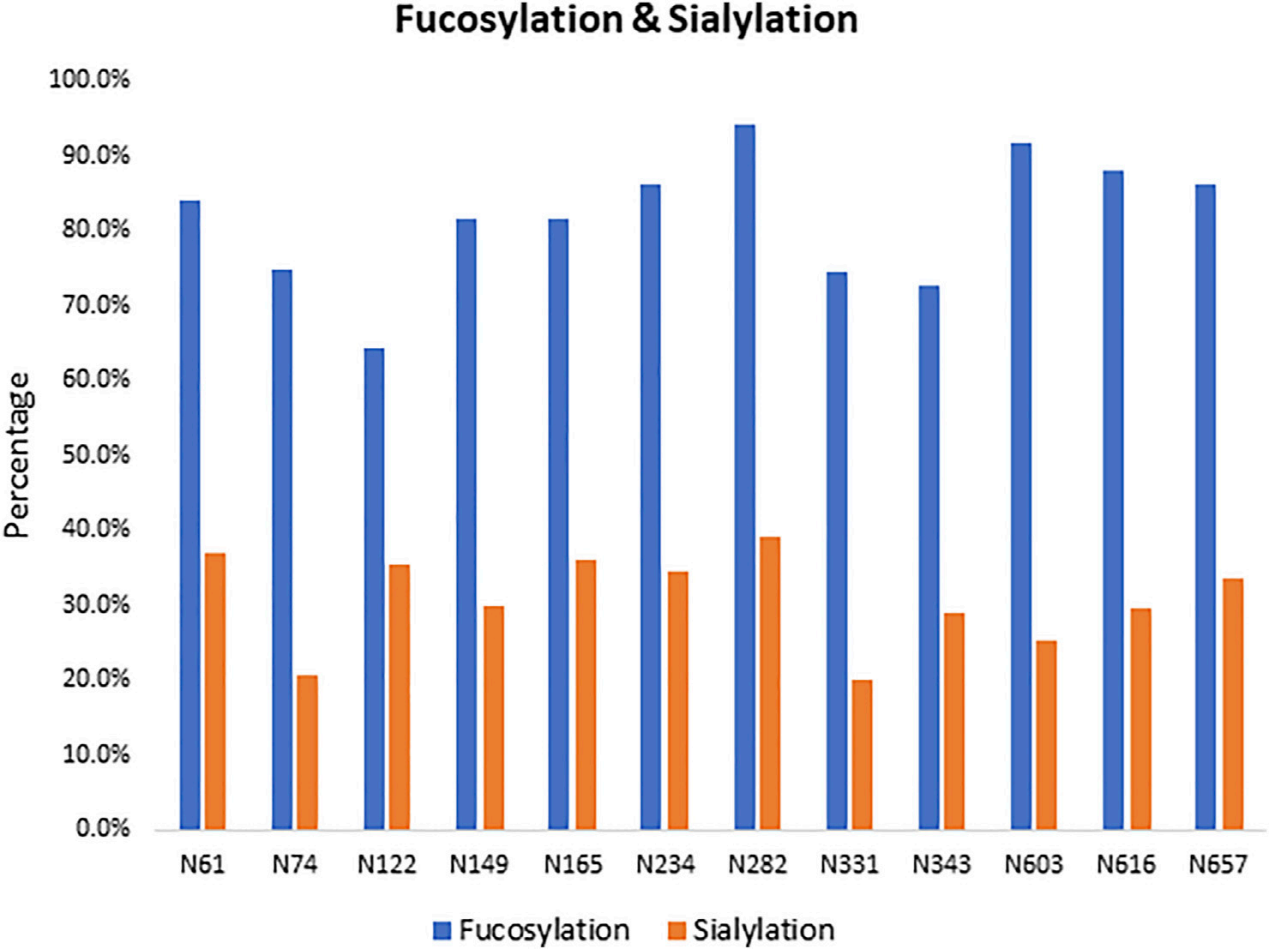
Fucosylated and sialylated N-linked glycosylation of the recombinant S1 protein. Twelve of 13 potential N-glycosylation sites were found occupied, and these N-linked glycans are highly fucosylated.

We observed a diverse range of glycan compositions across the N-linked glycosylation sites. Glycosites N331, N343, N603, and N616 had less glycan variety, while those at N122, N165, N234, N282, and N657 were more diverse (Supplementary Figure S3 and Supplementary Table S3). In addition to the site-specific glycan compositions, overall trends in glycosylation across sites were examined. The results revealed that the three most common types of N-glycans were Man5GlcNAc2 (Man5), HexNAc4Hex3Fuc1, and HexNAc5Hex3Fuc1 (Supplementary Figure S3). Man5 has also been reported by others as a predominant high-mannose glycan composition across all N-glycosites on the SARS-CoV-2 spike protein when produced in HEK293 cells but interestingly not in CHO cells (Zhao et al., 2020; Wang et al., 2021b). The relative abundance of complex-type glycans and the level of fucosylation and sialylation for each site were examined. As shown in Figure 2, the N-linked glycans on the S1 protein were both highly fucosylated (∼80%) and sialylated (∼30%) with overlap where both substitutions were observed on some glycans.

**FIGURE 3:**
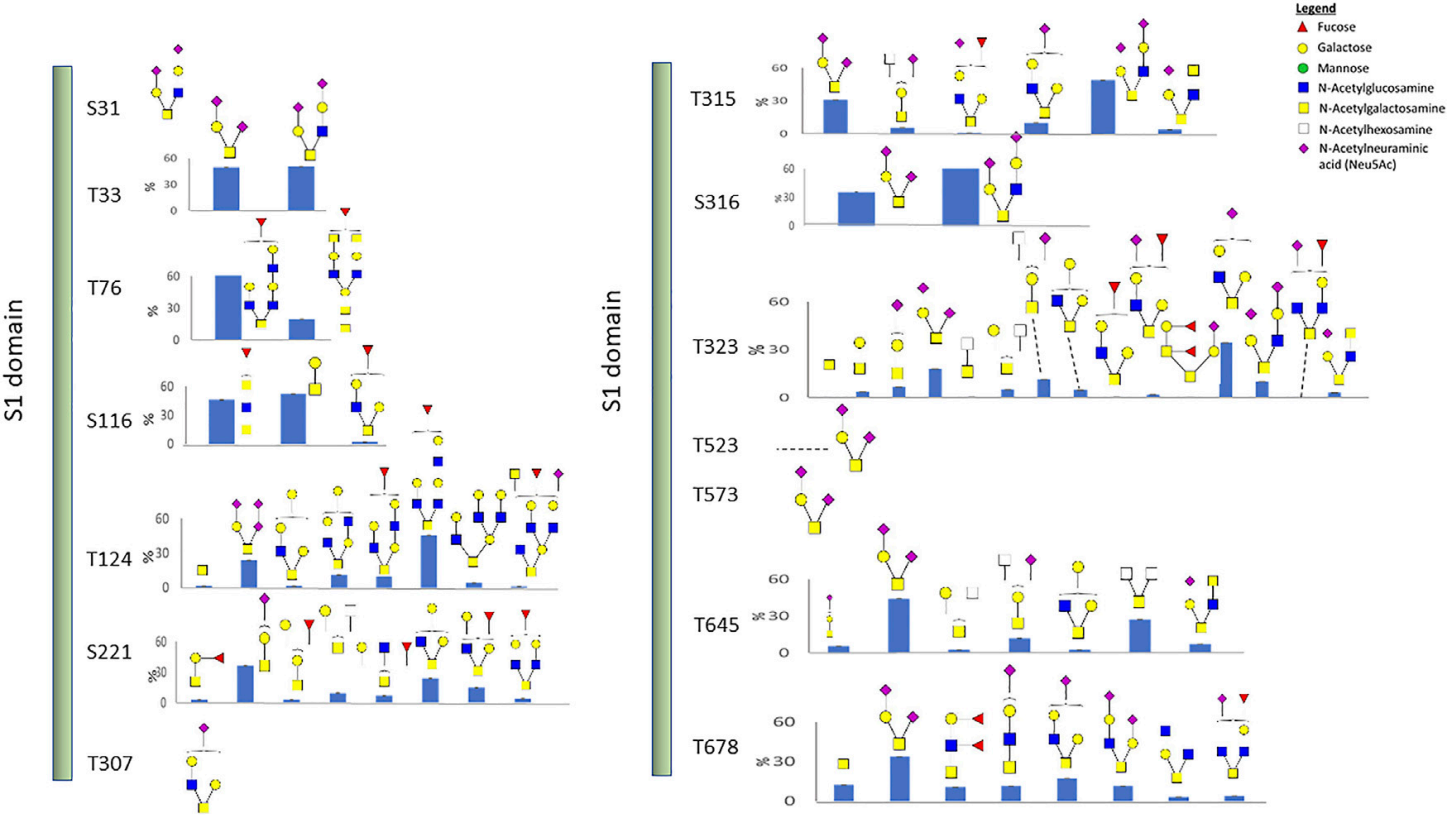
O-linked glycosylation of the recombinant S1 protein. Glycan cartoon structures are inferred from knowledge of common glycans as identification was solely based on mass.

We also identified 14 O-linked glycosites on the recombinant S1 protein, including the two sites, T323 and T523, which were identified in the recombinant RBD protein. O-glycosylation has been reported to function in immunological shielding, protein stability, and regulation of conformational changes (Casalino et al., 2020a). About half of the 14 sites have not been reported before, and most glycosites display a variety of glycan modifications (Figure 3 and Supplementary Table S4). Three O-glycosites, S673, T678, and S686, are located in the furin cleavage region. Thus, such glycans may modulate the SARS-CoV-2 entry (Andersen et al., 2020). Of the three hypothesized O-glycosites, T678 was identified in this study.

**FIGURE 4:**
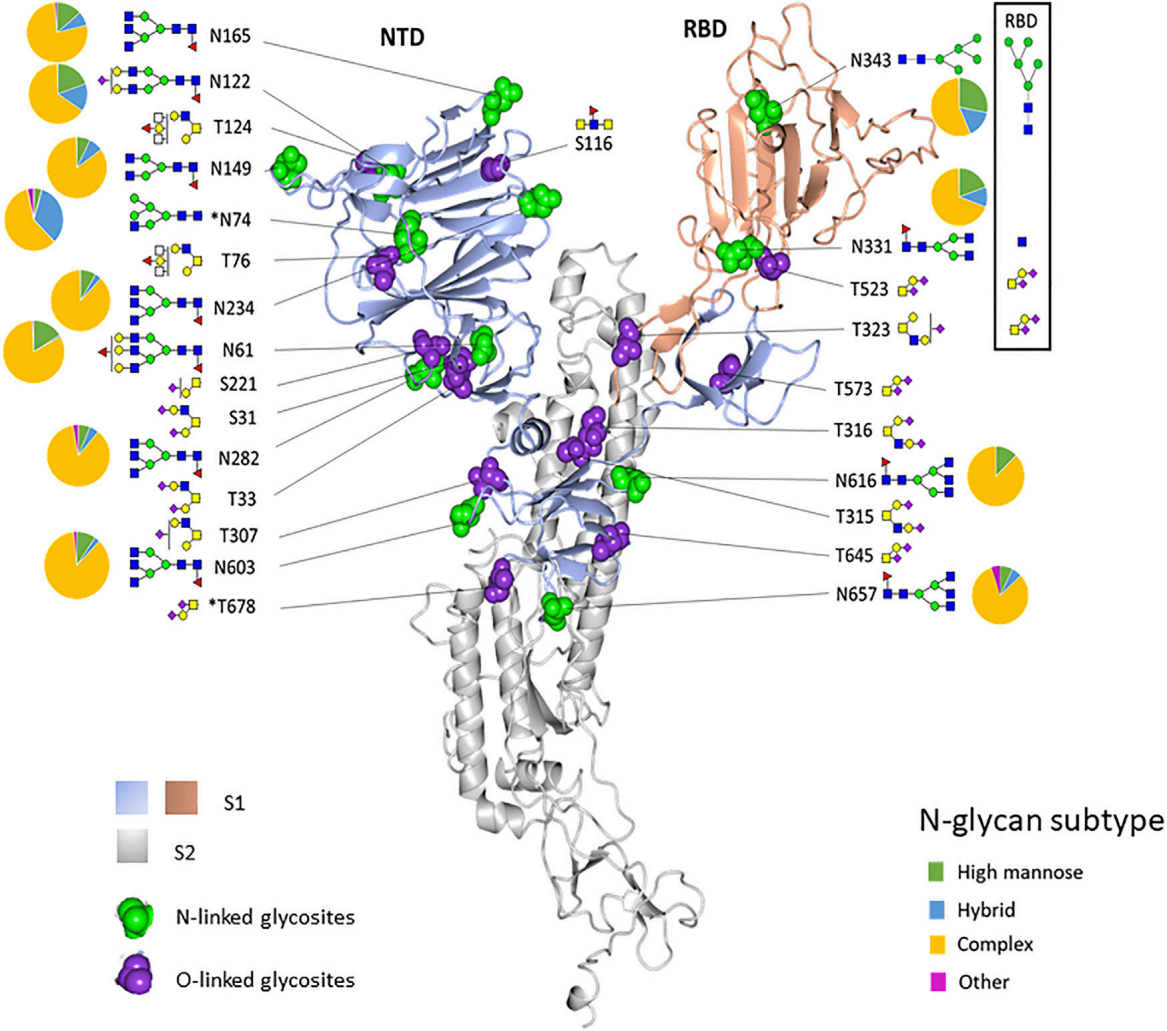
Structure-based mapping of glycans on the recombinant SARS-CoV-2 spike protein. The modeling of experimentally observed glycosylation site compositions is illustrated on the monomeric SARS-CoV-2 spike glycoprotein (PDB code 6ZGG). The S1 subunit is colored light blue and peach. The S2 subunit is gray. N- and O-linked glycosylation sites are indicated by green balls and purple balls, respectively. Most abundant glycans at each site are shown. Pie charts show the percentage of glycan subtypes at each site. The boxed area shows the predominant glycans and the N-linked glycosylation subtype distribution for the glycans identified in the recombinant RBD sample. *N74 and T678 are not in the structure.

The experimentally observed glycosylation sites are illustrated on the monomeric SARS-CoV-2 spike glycoprotein (PDB code 6ZGG) (Figure 4). To convey the main processing features at each site, the abundances of each glycan were summed by glycan subtype and displayed as a pie chart next to each site. We observed a combination of high-mannose, hybrid, and complex-type N-glycans for most of the sites. Overall, all glycosites were dominated by complex-type glycans when tabulated by subtype. N74 displayed more hybrid-type glycans (30%). N343, in the RBD region, displayed a higher relative amount of mannose-type glycans (28%). This observation aligned with our observations in the recombinant RBD protein (see Figures 1, 4).

**FIGURE 5:**
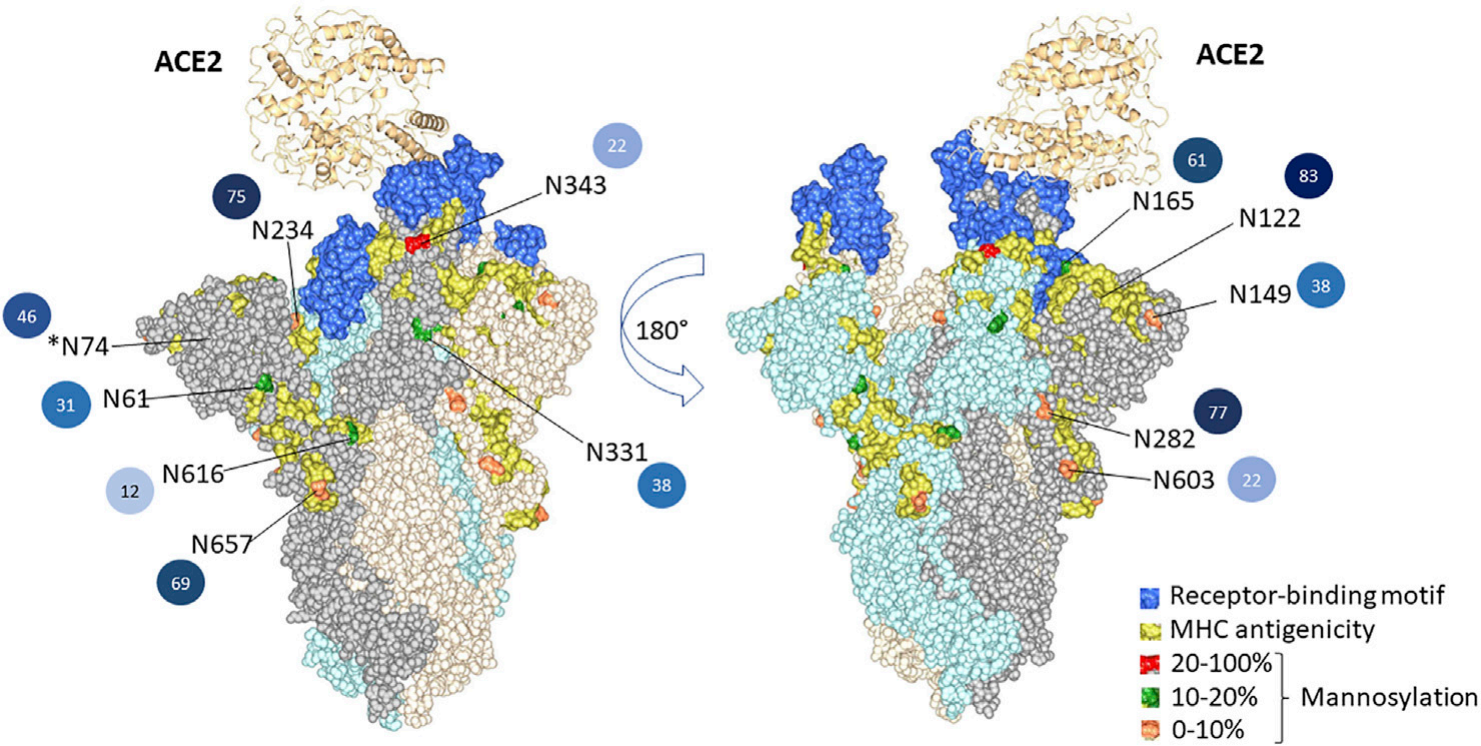
3D structural modeling of spike glycosylation microheterogeneity. The N-glycosites are mapped on the SARS-CoV-2 trimer structure (PDB code 7A96). Blue indicates the receptor-binding motif in the RBD region. Yellow indicates the predicted MHC antigenic sites. The glycosites are colored according to the mannosylation percentage. The number of glycoforms at each site from less to more heterogeneous glycoforms detected is colored by light to dark, and the number is also listed.

To illustrate the possible impact of the glycosylation microheterogeneity on the virus antigenicity, we mapped the N-glycosites with antigenic sites and the receptor-binding motifs to the SARS-CoV-2 trimer using a 3D model previously determined by electron microscopy (PDB code 7A96) (Figure 5). The data show extensive microheterogeneity across the glycosites. The number of identified glycoforms at each site ranged from 12 to 83. The antigenic epitopes were predicted using NetCTL-1.2 (Larsen et al., 2007) (Supplementary Table S5). We found that many occupied glycosites are close to, or even overlap with, the antigenic epitopes. Those that overlapped with antigenic sites included N165, N343, N616, and N657, which display substantial glycan diversity (Figure 5). The 3D model has one open RBD bound to the ACE2 protein. The shielding of receptor-binding sites by glycans is a common feature of viral glycoproteins and has been observed for the SARS-CoV spike, the HIV-1 envelope, and influenza hemagglutinin (Bonomelli et al., 2011; Stewart-Jones et al., 2016; An et al., 2019; Zhao et al., 2020).

There are two states of RBD: the “down” conformation and the “up” conformation, corresponding to the receptor-inaccessible state and receptor-accessible state, respectively (Gui et al., 2017; Walls et al., 2019; Wrapp et al., 2020). The modeling reveals that N343, N234, and N165 are near to the receptor-binding motif [limited to amino acids 438–506 (Zhou et al., 2021)]. Previous structure analysis revealed that in the RBD “down” state, the RBD region is shielded by the glycans at N343, N165, and N234 (Casalino et al., 2020b). Besides shielding, the glycans at N165 and N234 have also been reported to stabilize the RBD in the “up” conformation (Casalino et al., 2020b). Sztain et al. revealed that the receptor-binding motif is consistently shielded by the glycans at N165 and N234, but RBD opening decreases shielding by the glycans at N343 (Sztain et al., 2021). The N343 glycan may play the role of the “glycan gate” by facilitating conformational shift of the RBD from the “down” to the “up” conformation by interacting with the residues of the ACE2-binding motif (Sztain et al., 2021).

### Site-Specific Glycosylation of the Spike From the WA1 Strain and the D614G Variant

To determine the differences and similarities in glycosylation between the recombinant S1, produced in HEK293 cells, and that of the spike produced in the virus, we examined the spike derived from the intact virus from two strains, the WA1 strain and D614G, propagated in Vero E6 cells. The WA1 strain was from the first patient in the United States who was diagnosed with SARS-CoV-2 viral infection. This case was declared by the state of Washington and CDC on January 20, 2020 (Harcourt et al., 2020). This viral identity was confirmed by whole genome sequencing (GenBank accession no. MN985325), and it did not have mutation at the 614 amino acid. The D614G variant contains the spike protein amino acid change at 614 from D to G, which is more infectious and transmissible and has become the most prevalent form in the global pandemic since March 2021 (Hou et al., 2020; Korber et al., 2020). Both viruses were grown in Vero E6 cells, and the supernatant of the passage 4 stock of each virus was collected, inactivated by gamma irradiation, and analyzed by our glycoproteomics approach.

**FIGURE 6:**
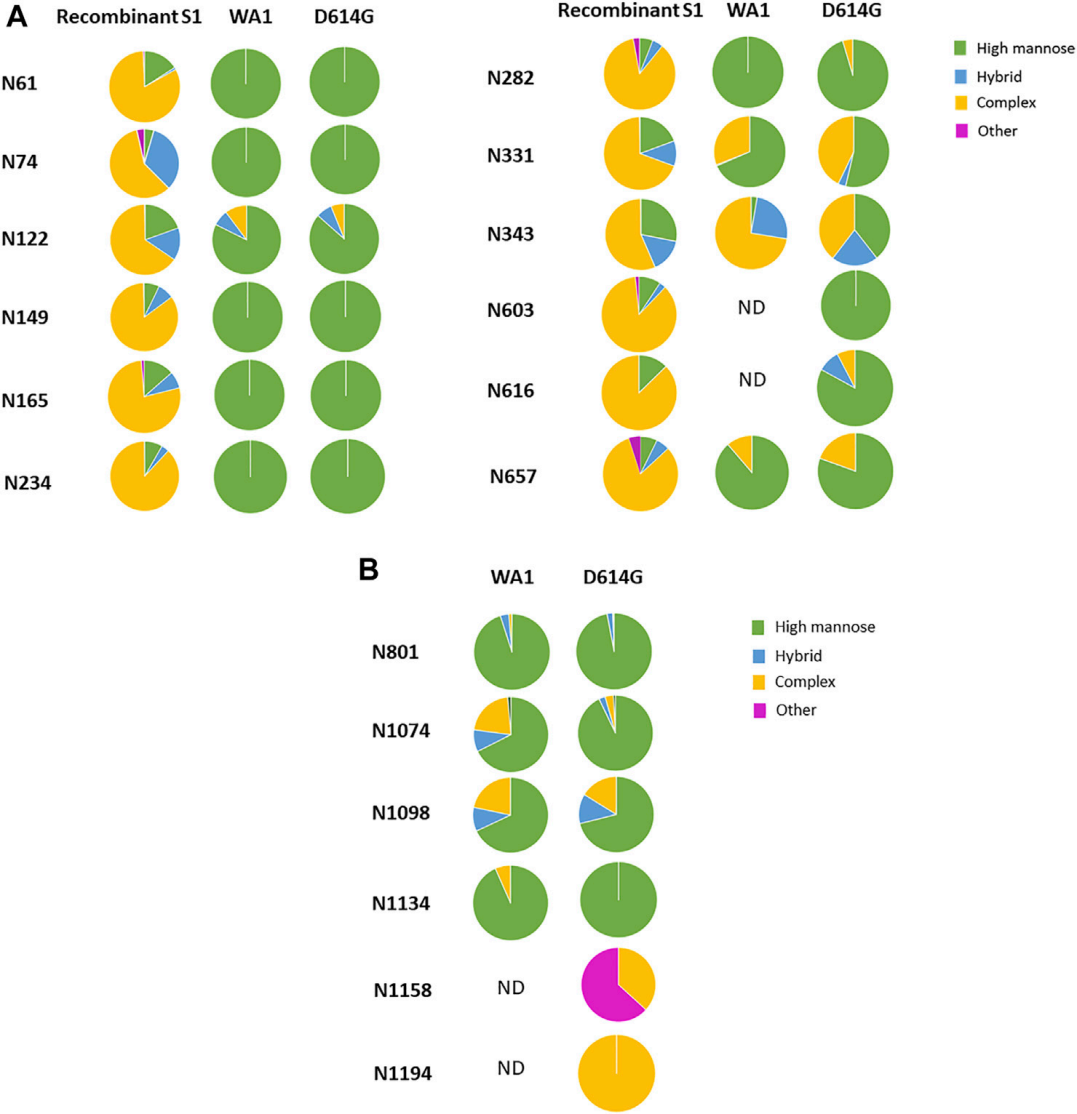
Comparison of the glycosylation pattern on the spike protein from recombinant S1, the WA1 strain, and the D614G variant. The most abundant glycoforms detected in Vero E6 cell-derived WA1 and D614G strain spike N-glycosites were comparable but different from that detected in recombinant S1 produced in HEK293. **(A)** N-linked glycan subtypes in the S1 domain. **(B)** N-linked glycan subtypes in the S2 domain.

Of the 13 predicted N-linked glycosites in the S1 domain, 10 N-glycosites were identified in the WA1 strain (Figure 6A and Supplementary Table S6). The two N-glycosites, N603 and N616, were identified with several high-mannose-type glycans (Man7GlcNAc2, Man8GlcNAc2, and Man9GlcNAc2 at N603 and Man8GlcNAc2 at N616) in a single replicate of the WA1 sample, which did not meet our criteria where two replicates were required to achieve confident identification. Therefore, glycosylation at these sites was considered tentative and not considered further. In contrast, 12 of 13 N-linked glycosites were identified in the S1 domain of the D614G variant (Figure 6A and Supplementary Table S7). Site occupancy identified by the PNGase F deglycosylation methodology revealed that 10 S1 N-glycosites from the WA1 strain (N61, N122, N165, N234, N282, N331, N343, N603, N616, and N567) and 9 N-glycosites from the D614G variant (N61, N149, N165, N234, N331, N343, N603, N616, and N657) were almost 100% glycosylated (Supplementary Tables S8, S9). Three N-glycosites, N74, N122, and N282, were only identified in D614G with a single replicate in the site occupancy study; therefore, we were not able to determine site occupancy at these three sites. (Supplementary Table S9). We do note, however, that these peptides are at least partially occupied by high-mannose glycans based on our glycopeptide analysis (Figure 6A and Supplementary Table S7). Likewise, some N-glycosites were identified with highly diverse glycan compositions upon glycoproteomics analysis of intact glycopeptides. However, occupancy analysis at sites such as N149 in the WA1 strain and N122 in D614G strains did not meet our criteria. Conversely, no glycopeptides were identified at N603 and N616 of the WA1 strain, but these two sites were identified as occupied based on detection of Asp in place of Asn subsequent to PNGase F digestion, which supports that the two sites were glycosylated. Estimated occupancies were between 96.4 and 100%, making it unlikely that spontaneous deamination of unoccupied sites was solely responsible for the Asp presence at the site. The most likely reason for the discrepancy is due to the random sampling issue of mass spectrometry or incomplete enrichment for these very complex samples. The samples of intact viruses contained less than 5% of spike protein abundance according to the protein quantitation by Byonic software. High-abundance glycopeptides of host cells were also enriched using the HILIC column, which resulted in the high complexity of the glycopeptide pool in this experiment.

**FIGURE 7:**
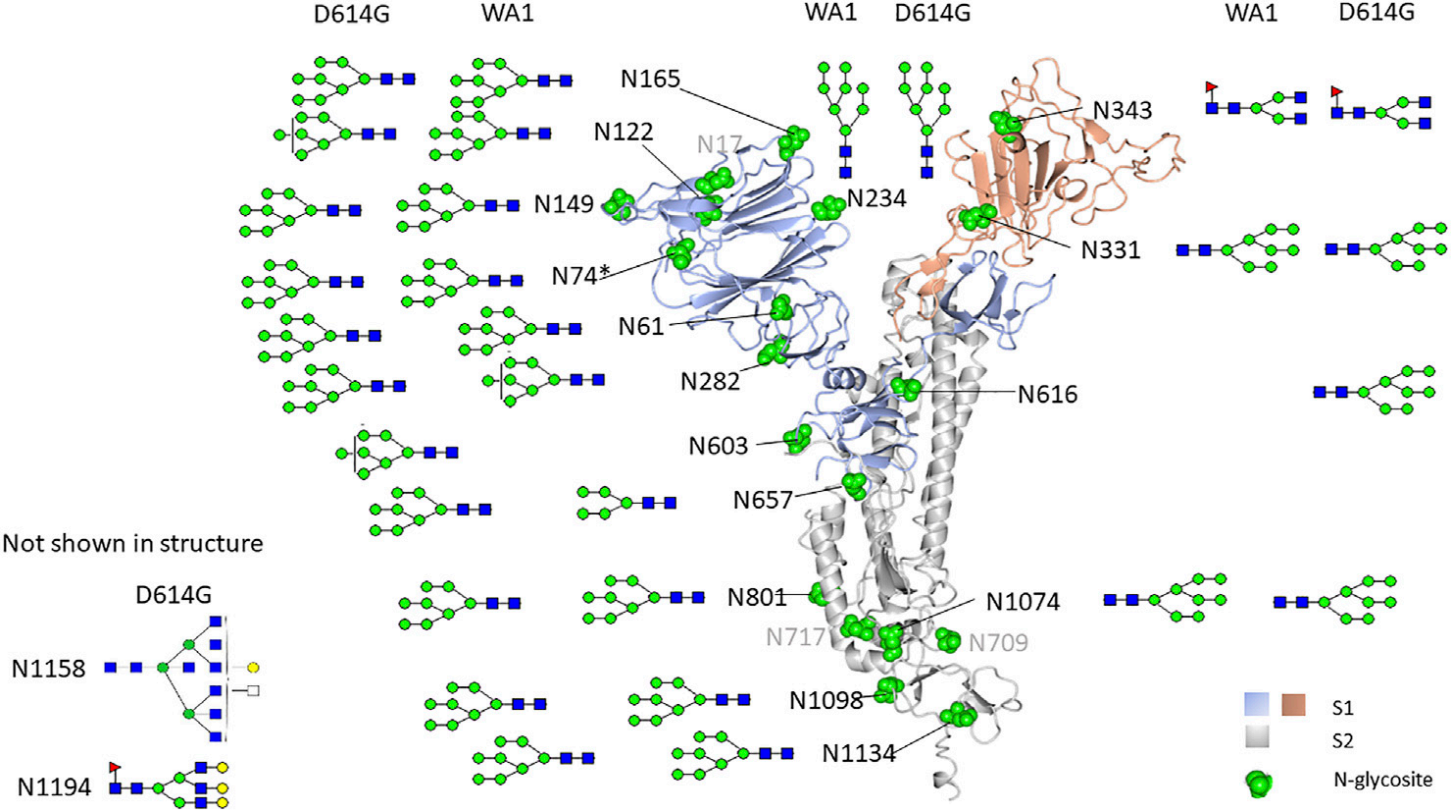
N-glycosylation map of Vero E6-derived viruses. The most abundant glycoform detected in Vero E6 cell-derived WA1 and the D614G strain spike N-glycosite was comparable but different from that detected in recombinant S1 produced in HEK293.

When comparing the mutant form, D614G, with the original form, WA1, we observed a similar glycosylation pattern for most N-linked glycosites in both S1 and S2 domains (Figures 6A,B). The most abundant glycoform at each N-glycosite was comparable between WA1 and D614G (Figure 7). Man7GlcNAc2 and Man8GlcNAc2 were the most abundant glycoforms for the majority of the sites, except for N343. There are several sites showing different glycan contents between the two strains, such as N331, N343, and N1074. The D614G variant presents more complex-type glycans at N331 but less complex-type glycans at N343 compared to the WA1 strain. As mentioned earlier, the N343 glycans significantly affect the RBD “up” conformation (Sztain et al., 2021). The glycan changes at N343 in D614G compared to WA1 could, at least partially, account for D614G phenotype changes if similar shifts in glycosylation occur in nature.

In addition, four glycosites, N603, N616, N1158, and N1194, were not identified in the WA1 strain, while all four were identified in the D614G variant (Figures 6A,B, Supplementary Tables S6 and S7). This may not mean that these sites are not glycosylated in WA1. Their absence may have resulted from sample complexity, random sampling, and limitations of our enrichment strategy as discussed above. This hypothesis is supported by the site occupancy analysis where WA1 spike N603, N616, and N1194 sites were clearly occupied (Supplementary Table S8).

Interestingly, in the recombinant S1 protein, most N-linked glycosites are dominated by complex-type N-glycans, while most glycosites in both the WA1 strain and the D614G variant produced in the virus were dominated by high-mannose-type glycans (Figure 6A). No O-linked glycosites were identified in the virus-derived spike from WA1 and D614G. The reason for the observed dramatic differences in the glycosylation pattern detected between recombinant spike S1 and the virus-derived spike is not clear. There are several possibilities: first, protein structural differences may influence access to glycosylation machinery. Second, the expression of glycosylation enzymes may differ between cell substrates. Third, the secretory location of glycosylation enzymes may differ between cell substrates under conditions of expression and/or virus propagation. WA1 and D614G strains were grown in Vero E6 cells which are derived from the African green monkey kidney, while the recombinant S1 protein was expressed by HEK293 cells which are derived from human embryonic kidney cells.

It is clear that S1 and the whole spike differ structurally as the former is without the S2 subunit. Several publications reported differences in glycosite occupancy and glycan composition between the intact spike protein and individually expressed S1 and S2 proteins (Watanabe et al., 2020a; Watanabe et al., 2020b; Shajahan et al., 2020; Zhao et al., 2020). The spike expressed in FreeStyle293F cells was found to be partially expressed as the S0 form, without S1/S2 or S2′ cleavage. The S0 form was found to primarily contain high-mannose glycans (Watanabe et al., 2021). We searched our data for evidence of S1/S2 cleavage. We did detect cleavage of the S1/S2 furin cleavage site in the chymotrypsin digest. However, the intensity of these peptides was low, suggesting that significant amounts of the uncleaved S0 forms were present. We must also note that no peptides representing the uncleaved site were detected. It has been reported that Vero E6 cells do not produce high-abundance furin and cleavage of the S glycoprotein in SARS-CoV-2-infected Vero E6 cell lysates was reported to be inefficient (Klimstra et al., 2020). One study, using human serum to detect SARS-CoV-2 proteins produced in infected Vero E6 cell lysates, showed mainly an uncleaved S protein (Haveri et al., 2020). Additionally, in our case, there was also no evidence for TMPRSS2 or cathepsin cleavage. Our observations, however, may have been due to low abundance of the spike in our samples; thus, peptides specifically containing these cleavage sites may not have been detected.

In general, Vero cells are not known to limit glycan processing primarily to high-mannose glycans. Vero cells have been used as a cell substrate for propagation of influenza and recombinant proteins without report of bias toward high-mannose glycans (Gornik et al., 2012; Rödig et al., 2013). The nascent capacity of Vero cell expression and the secretory location of glycosylation enzymes should not be an issue. Therefore, our observations are not likely to be due to inherent limitations of Vero cells in terms of glycosylation processes. However, our data show that high-mannose-type glycans represent a large portion of total glycans displayed on the Vero E6 host’s glycoproteins (Supplementary Figure S4). A range of complex glycans were also identified, albeit with far less abundance. Therefore, the high percent of high-mannose-type glycans on WA1 and D614G grown in Vero E6 cells was not limited to the SARS-CoV-2 spike.

The proper location of glycosylation enzymes is a complex process involving Rab GTPases, coiled-coil tethers termed golgins, and the multi-subunit tethering complex known as the conserved oligomeric Golgi (COG) complex (Fisher and Ungar, 2016). These factors contribute toward anterograde and retrograde transport of glycosylation active enzymes and other necessary proteins involved in glycoprotein production. Regulation of these processes is essential for appropriate localization and sequential activities of glycosylation active enzymes (Starr et al., 2010). In our studies of the Vero cell-propagated SARS-CoV-2 spike, we noted a low amount of glycosylation processing beyond ER mannosidase I (Moremen et al., 2012) and other mannosidases which are normally present in the cis/medial cisternae of the Golgi (Moremen et al., 2012). This was evidenced by the dominant presence of primarily Man5-8GlcNAc2. There were only low abundances of Man3-5GlcNAc3, also suggesting little processing by cis/medial Golgi located N-acetyglucosaminidase I (Gnt1) (Sztain et al., 2021; Moremen et al., 2012). Therefore, one possibility is that under conditions of viral propagation, the Golgi COG system-mediated anterograde/retrograde system is shifted or viral packaging and routing differs from normal secretion, resulting in an altered distribution of glycosylation active enzymes or proper sequential exposure of these enzymes to nascent glycoproteins. Notably, virus-like particles and the SARS-CoV-2 virus have been localized to the endoplasmic reticulum–Golgi intermediate compartment (ERGIC), a site of secretory sorting between the ER and Golgi, and it has been hypothesized that SARS-CoV-2 exits the cell *via* lysosomal exocytosis, suggesting little exposure to Golgi enzymes (Mendonça et al., 2021; Plescia et al., 2021). We note that among 25 cell lines tested, Vero E6 produced among the highest viral titers including all those expressing the human ACE2 receptor. Therefore, the high-mannose glycan distribution does not appear to significantly negatively affect viral propagation in the Vero E6 cell line compared to alternative cell substrates typically used in the SARS-CoV-2 viral study (Wang et al., 2021a).

Watanabe et al. (2021), who also noted unprocessed glycans on the spike, albeit produced in HEK cells expressing the ChAdOx1 vaccine vector, hypothesized that these high-mannose-bearing spike proteins represented those in transit through the secretory system and suggested that furin protease is located in the later trans-Golgi stacks. In our case, this is unlikely as the majority of the virus isolated formed mature viral particles. Significantly, both HEK293 and Vero cells produce predominantly high-mannose glycosylation patterns on the SARS-CoV-2 spike under certain circumstances. The exact reason for this remains an open question.

Overall, we have found similar glycosylation site-specific N-glycan distributions in S1 and RBD to those previously reported that were produced in HEK293 cell lines. We also report here previously unreported O-glycosylation site occupancy including T523 and confirm the presence of 14 total O-glycosylation site occupancies including T678, which appears in the furin cleavage domain. Significantly, we also report that the native spike produced in SARS-CoV-2/USA-WA1/2020 (USA-WA1) is substituted with primarily high-mannose glycans that do not appear to effect viral propagation in Vero E6 compared to alternative cell substrates (Wang et al., 2021a).

## Conclusion

In this study, we characterized the site-specific glycosylation of the spike protein from recombinant RBD and S1 domains and from two intact viruses, the WA1 strain and the D614G variant. Glycosylation was found to be of high occupancy in all samples examined and highly heterogeneous across the majority of glycosites in the HEK293-derived S1 and RBD. Glycan modification at most N-glycosites is very similar between WA1 and D614G and primarily high-mannose, with significant differences at N343. Our results also revealed different patterns of glycan modification among the recombinant S1 protein, recombinant RBD, and the WA1 and D614G strains, which implies that these spike proteins may perform differently *in vitro* and *in vivo*. Therefore, the origin of spike glycosylation should be put in consideration for vaccine design and drug development.

## Author Summary

The SARS-CoV-2 virus spike protein binds to host cells, fuses with the host cell membrane, and enters the cell. It is heavily glycosylated, and recent studies revealed that glycan modification is essential to modulate spike conformation and host cell invasion. In this study, we analyzed the glycan modification of recombinant spike protein subunit RBD and the S1 domain, both of which function to bind host receptor ACE 2. We also analyzed the glycan modification of whole viruses, the WA1 strain, and the D614G variant. The WA1 strain was isolated from the first case of COVID-19 in the United States. The D614G variant, carrying the protein amino acid change at 614 from aspartate(D) to glycine(G), is now prevalent in the circulating SARS-CoV-2 virus and is carried by all recently identified and highly concerning SARS-CoV-2 variants. We found different patterns of glycan modification among the recombinant S1 protein, recombinant RBD, and WA1 and D614G strains. Glycan modification at most N-glycosites is very similar between WA1 and D614G, with significant differences at N343. This recombinant S1 and RBD glycosylation patterns differ dramatically from the whole virus produced in Vero cells and implies that these spike proteins may perform differently *in vitro* and *in vivo*, which could have implications for viral studies, vaccine design, and drug development.

## Data Availability

The datasets presented in this study can be found in online repositories. The names of the repository/repositories and accession number(s) can be found below: ProteomeXchange Consortium via the PRIDE partner repository with the dataset identifier PXD029218.

## Supplementary Material

Supplementary Figure S1 Spectra of glycopeptides with short complex glycans identified at N331 from the recombinant RBD protein.

Supplementary Figure S2 Spectra of O-linked glycopeptides identified at T523 from the recombinant RBD protein.

Supplementary Figure S3 Relative abundances of all N-linked glycans detected at each site of the recombinant S1 protein. Twelve of 13 potential N-glycosylation sites were found occupied and are displayed. The displayed value is the mean abundance of each glycan detected across three biological replicates normalized to total ion abundance at each site.

**Supplementary Figure S4** | Glycan profiles identified from glycoproteins of Vero E6 cells. **(A)** N-glycans identified from WA1 trypsin and the GluC digest. **(B)** N-glycans identified from the WA1 chymotrypsin digest.
