# Supplementary material for: Site-Specific Glycosylation Patterns of the SARS-CoV-2 Spike Protein Derived From Recombinant Protein and Viral WA1 and D614G Strains
Source: Front Chem. 2021 Nov 19;9:767448. doi: 10.3389/fchem.2021.767448 (PMC8640487; doi:10.3389/fchem.2021.767448)
Supplement: Supplementary file 1 [file Presentation1.PPTX]

## Slide 1
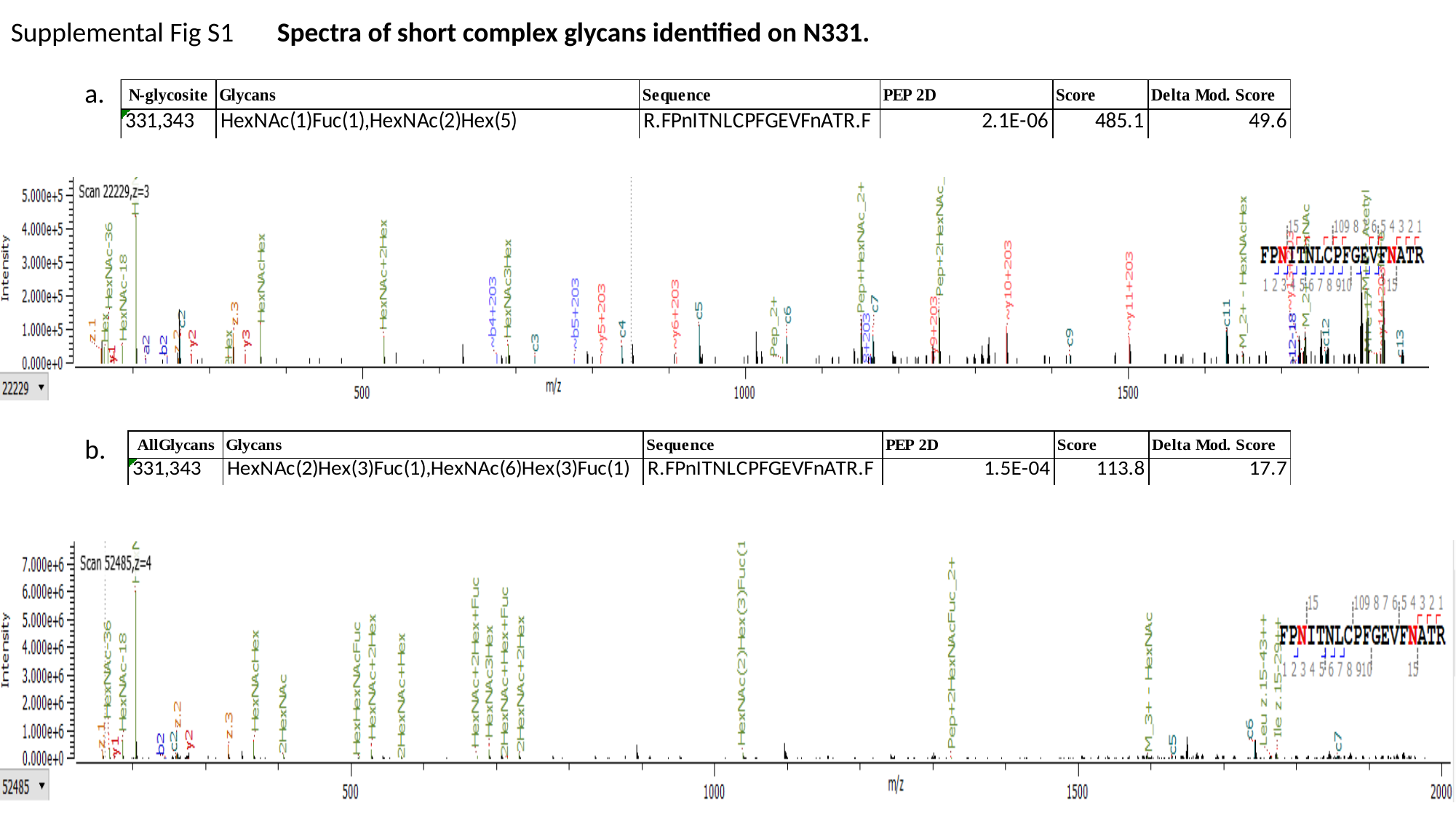

Supplemental Fig S1
Spectra of short complex glycans identified on N331.
a.
b.

## Slide 2
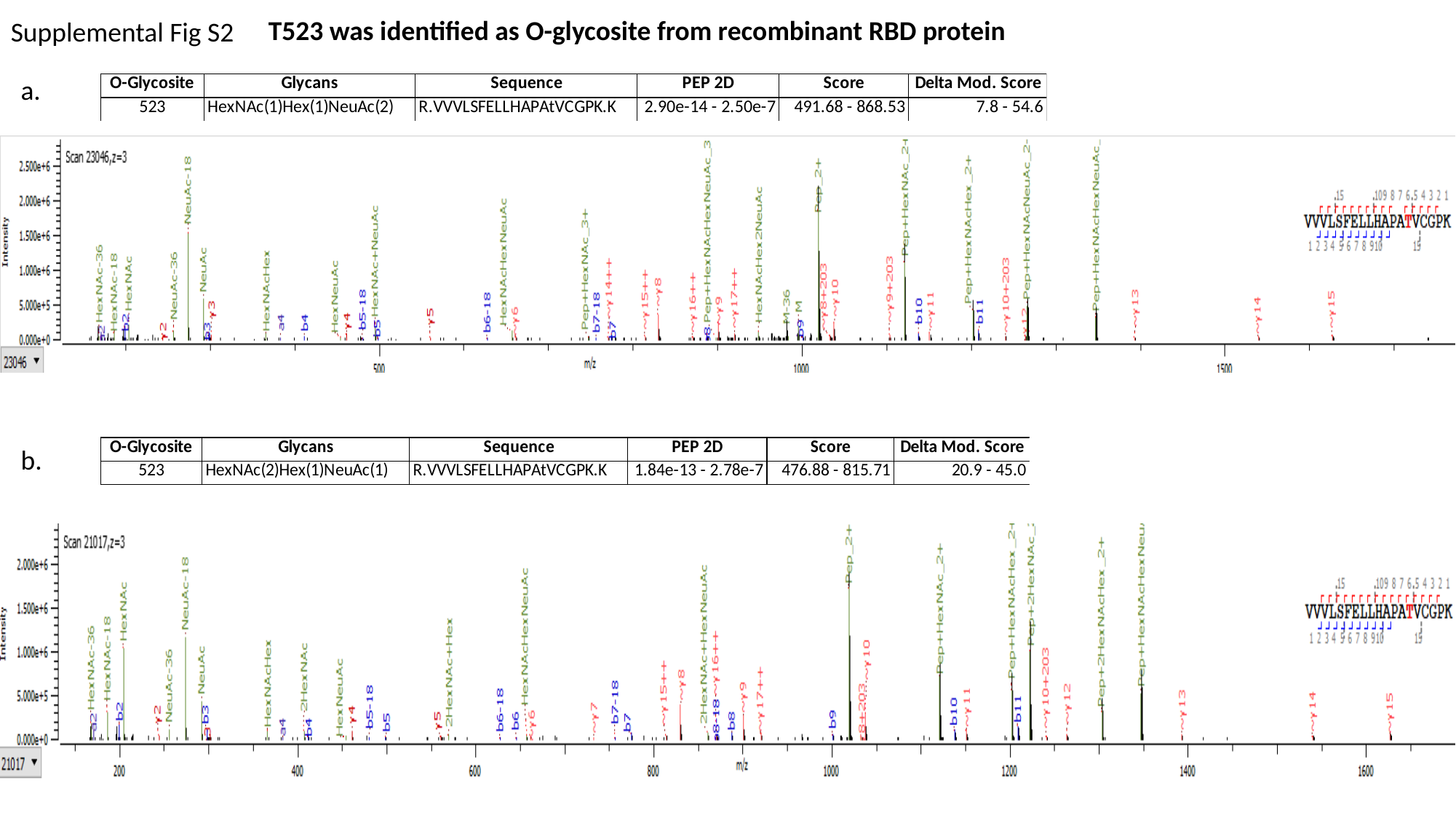

T523 was identified as O-glycosite from recombinant RBD protein
Supplemental Fig S2
a.
b.

## Slide 3
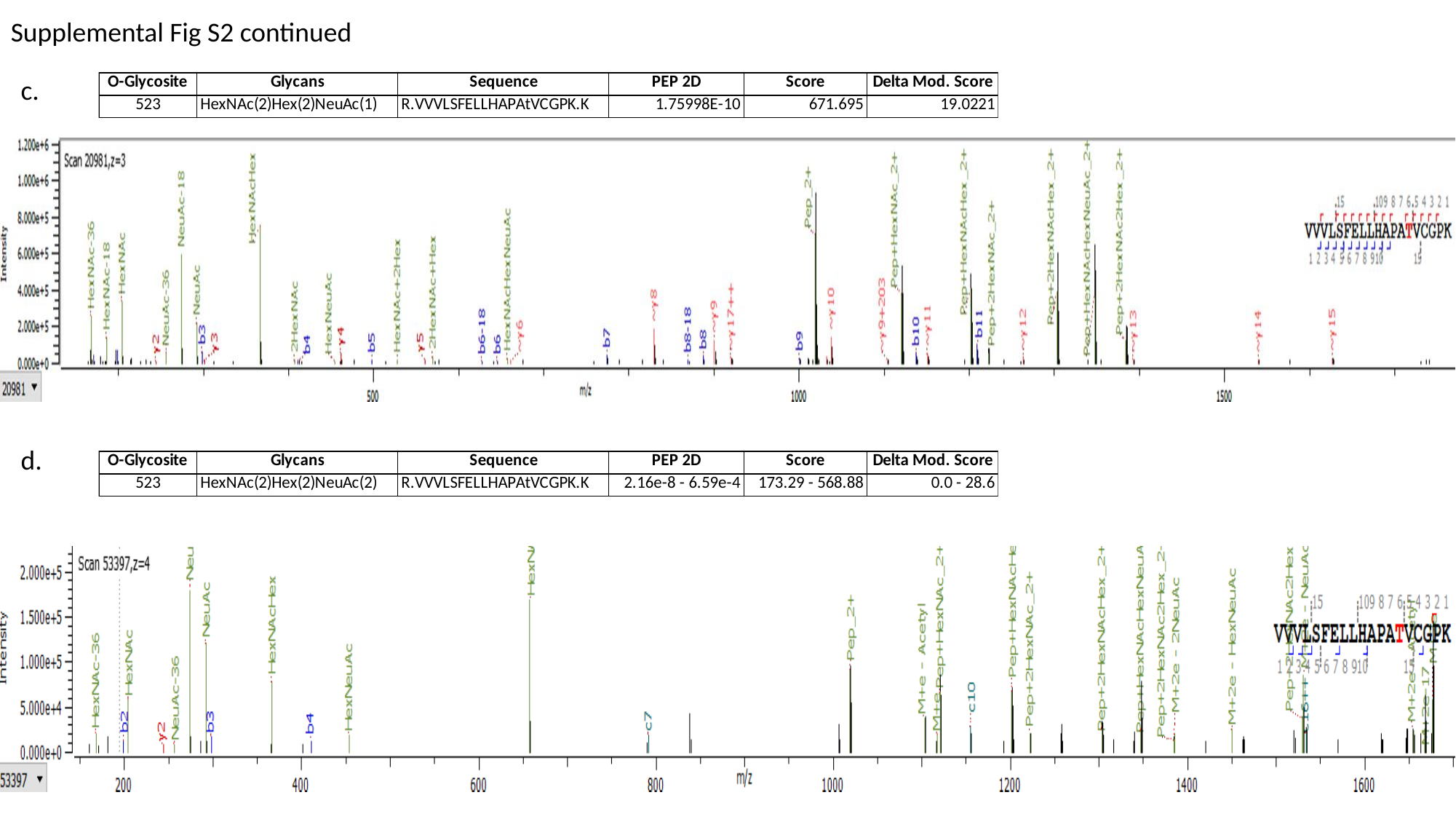

Supplemental Fig S2 continued
c.
d.

## Slide 4
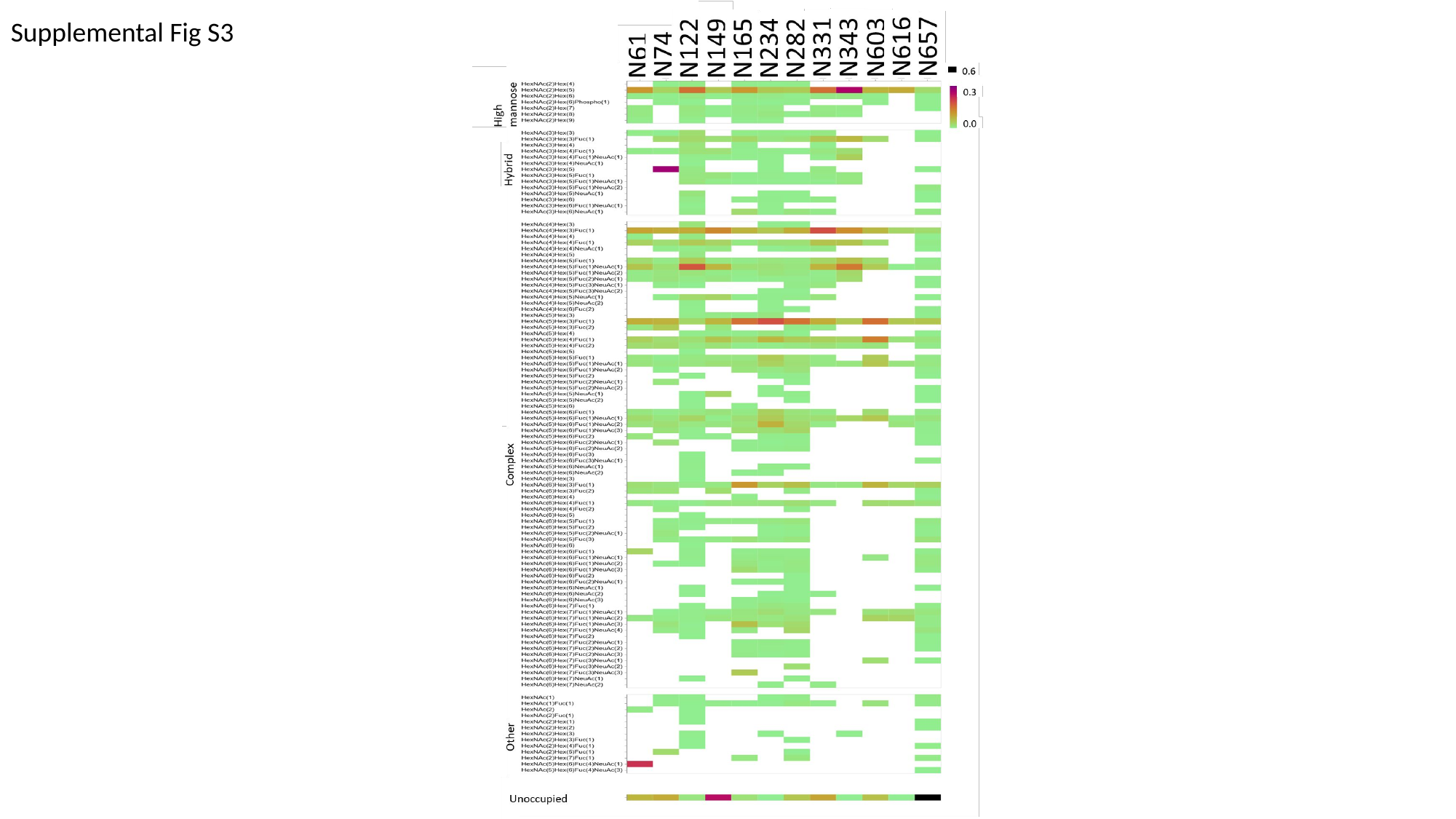

Supplemental Fig S3

## Slide 5
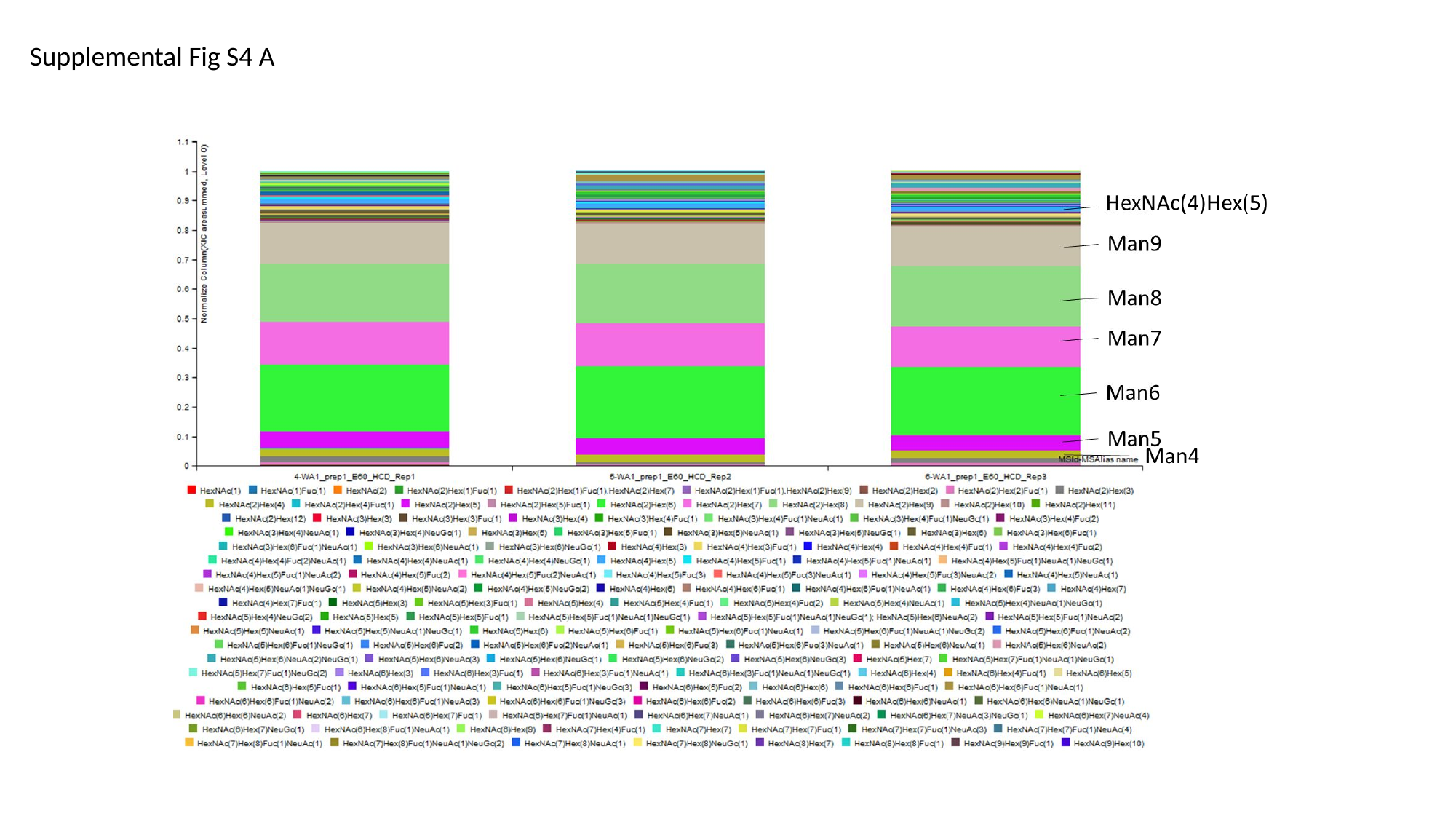

Supplemental Fig S4 A

## Slide 6
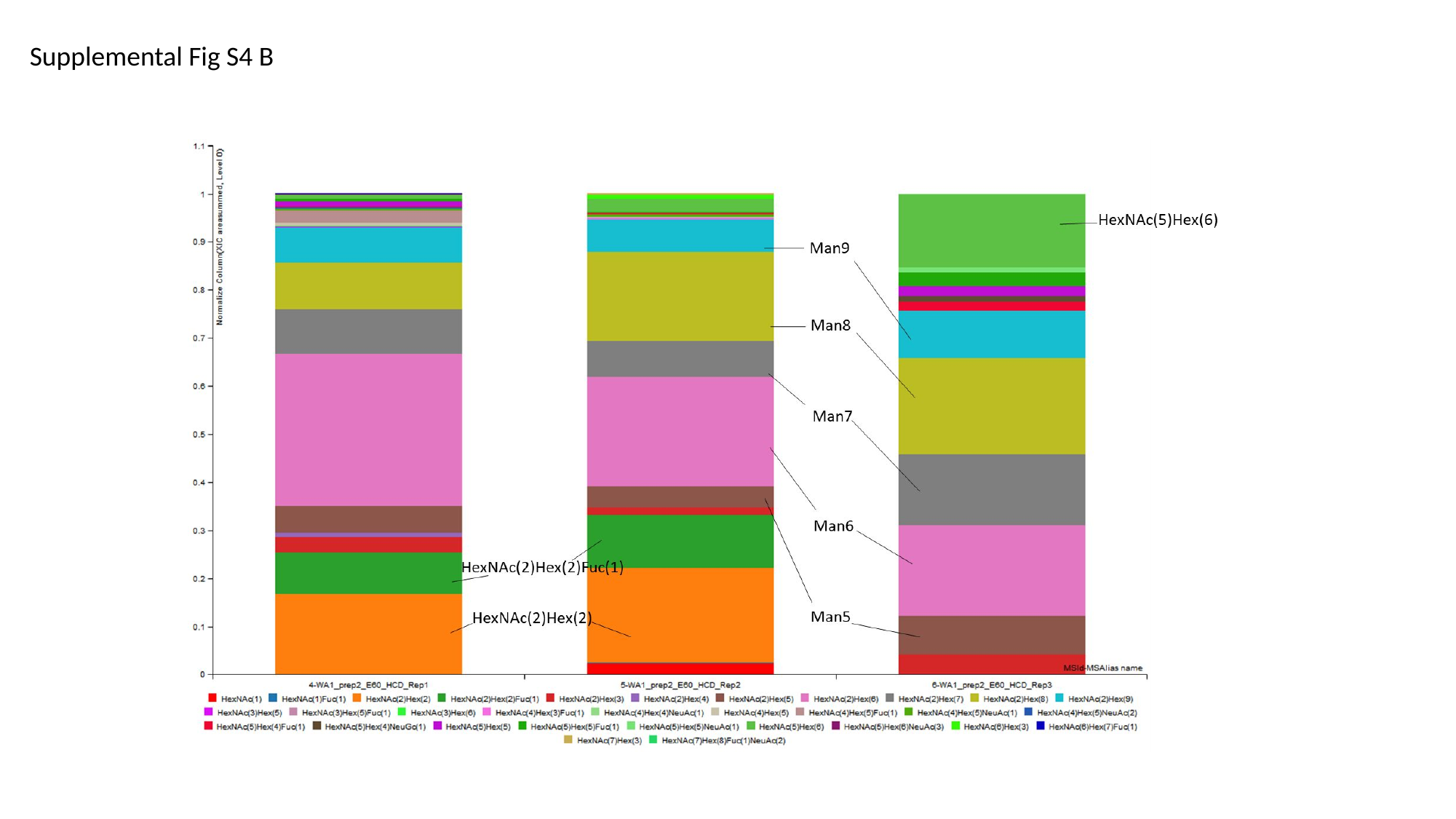

Supplemental Fig S4 B
